# Predicting dichotomised outcomes from high-dimensional data in biomedicine

**DOI:** 10.1080/02664763.2023.2233057

**Published:** 2023-07-26

**Authors:** Armin Rauschenberger, Enrico Glaab

**Affiliations:** Luxembourg Centre for Systems Biomedicine (LCSB), University of Luxembourg, Esch-sur-Alzette, Luxembourg

**Keywords:** Linear/logistic regression, numerical prediction, binary classification, dichotomisation, high-dimensional data, ridge/lasso regularisation, 62-04, 62J07, 62J05, 62H30, 62P10

## Abstract

In many biomedical applications, we are more interested in the predicted probability that a numerical outcome is above a threshold than in the predicted value of the outcome. For example, it might be known that antibody levels above a certain threshold provide immunity against a disease, or a threshold for a disease severity score might reflect conversion from the presymptomatic to the symptomatic disease stage. Accordingly, biomedical researchers often convert numerical to binary outcomes (loss of information) to conduct logistic regression (probabilistic interpretation). We address this bad statistical practice by modelling the binary outcome with logistic regression, modelling the numerical outcome with linear regression, transforming the predicted values from linear regression to predicted probabilities, and combining the predicted probabilities from logistic and linear regression. Analysing high-dimensional simulated and experimental data, namely clinical data for predicting cognitive impairment, we obtain significantly improved predictions of dichotomised outcomes. Thus, the proposed approach effectively combines binary with numerical outcomes to improve binary classification in high-dimensional settings. An implementation is available in the R package cornet on GitHub (https://github.com/rauschenberger/cornet) and CRAN (https://CRAN.R-project.org/package=cornet).

## 1. Introduction

Many diagnostic and prognostic problems in biomedicine are essentially binary classification tasks. A binary outcome splits samples into two groups of interest. Some binary outcomes are *naturally* binary, whereas other binary outcomes are *artificially* binary [30]. We focus on artificial binary outcomes that result from the dichotomisation of numerical outcomes with a single threshold. Such binary variables indicate whether the underlying measurements are greater than a given cut-off value.

While there are strong reservations against outcome dichotomisation in the statistical literature [3,8,15,16,20,27], it remains popular in empirical research. The main problem for prediction is that dichotomising a numerical outcome implies a loss of information equivalent to discarding a certain proportion of the data [2], although it might simplify the understanding and communication of results [6,7] or increase robustness against contamination [26]. In our experience, researchers often underestimate the disadvantages or overestimate the advantages of dichotomisation.

However, many biomedical applications require predicted probabilities rather than predicted values. Suppose there is a critical transition if *y*>*c*, where *y* denotes a clinical outcome, and *c* denotes a threshold. Then we would want to predict P(y>c) rather than *y*. Typically, the prediction y=c means that the probability of the critical transition is about 50%, but other predictions y≠c) are more difficult to interpret, because they only tell whether the probability is below or above 50%. Even if the threshold is only an estimate of the tipping point where the critical transition occurs, we might want to predict the probability that the outcome will exceed this threshold, e.g. to make or to predict a treatment decision. (This also holds for *arbitrary* thresholds. Suppose a clinical protocol requires mechanical ventilation if the oxygen level falls below a certain value: Even if the patient could cope with lower values, we might want to predict whether the physician will use a ventilator.)

The analysis of modern biomedical data, typically including some hundred samples but many thousand features, requires new statistical methods. In this paper, we propose an approach to obtain improved predictions of dichotomised outcomes in high-dimensional settings (i.e. settings with many more features than samples).

The same problem has previously been addressed in low-dimensional settings [5,14,29] (i.e. settings with many fewer features than samples). Although the proposed approach is novel, we consider these and other related methods for possible extensions (see Section 5). There are methods that address different problems but also combine binary and numerical outcomes, such as risk estimation for dichotomised outcomes [28], bivariate regression for binary-continuous outcomes [4,9], odds ratios for linear regression [18], and ordinal logistic regression [13]. A recurrent idea is to exploit information from the numerical outcome and provide interpretation for the binary outcome.

This manuscript describes a straightforward approach to predict dichotomised outcomes from high-dimensional data. A more complex predictive method (e.g. random forests or neural networks for obtaining predicted values) together with a calibration method (e.g. Platt scaling or isotonic regression for transforming predicted values to predicted probabilities) could provide more predictive models, but these would be less interpretable (‘black box’). We solve this specific prediction problem by combining two familiar methods (linear and logistic regression with lasso or ridge regularisation), leading to models that are not only predictive but also interpretable.

## 2. Approach

### 2.1. Overview

Our goal is to predict the (artificial) binary outcome, rather than the (natural) numerical outcome, from many features. For any sample, we either know or ignore both outcomes. Our strategy is to learn from the samples with observed outcomes how the features affect *both* outcomes, in order to predict the *binary* outcome of the samples with unobserved outcomes. A challenge in supervised learning (especially in high-dimensional settings) is to avoid overfitting, which occurs if the model fits well to the observed data but not to unobserved data. This is how we model the two outcomes based on many features:

- two outcomes: In the generalised linear model framework, a suitable approach for binary outcomes is logistic regression, and a suitable approach for numerical outcomes is linear regression. Given both types of outcomes, we can fit both regression models. In most cases, linear regression is the better choice, because the numerical outcome is normally more informative than the binary outcome (see Examples 1 and 2 in Section 3.3). In some cases, however, logistic regression is the better choice, because it is more robust against departures from linearity and normality (see Examples 3 and 4 in Section 3.3). While logistic regression returns predicted probabilities, linear regression returns predicted values.
- many features: In low-dimensional settings without strong multicollinearity, we could estimate the regression coefficients by maximising the likelihood function. But in high-dimensional settings, which include many more features than samples, we need to regularise the regression coefficients. The lasso and ridge penalties, whose weighted sum is the elastic net penalty [33], increase with the absolute or squared values of the coefficients, respectively. Both penalties shrink the coefficients towards zero (regularisation), but only the lasso penalty sets coefficients equal to zero (variable selection).

**Figure 1.**
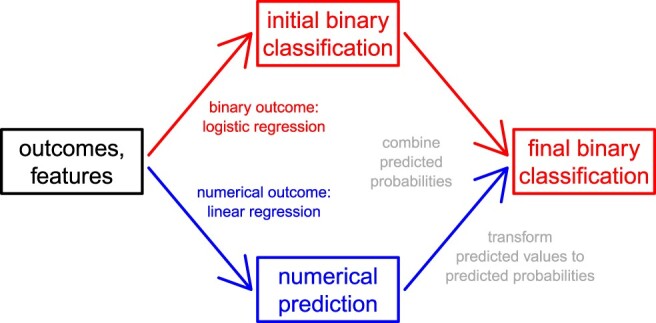
After modelling the (artificial) binary outcome with penalised logistic regression, and the (original) numerical outcome with penalised linear regression, we use the numerical prediction to improve the binary classification.

We combine logistic and linear regression, with lasso or ridge regularisation, to predict dichotomised outcomes. This leads to two estimated effects for each feature, one from logistic regression and one from linear regression, and two predictions for each sample, one from logistic regression and one from linear regression. The proposed approach transforms the predicted values from linear regression to predicted probabilities and combines these predicted probabilities with those from logistic regression. Figure 1 illustrates the workflow.

### 2.2. Data

We observe one numerical outcome and *p* features for *n* samples. Let *i* in {1,…,n} index the samples, and let *j* in {1,…,p} index the features. For each *i* and *j*, let $y_{i}$ denote the outcome for sample *i*, and let $x_{\mathrm{ij}}$ denote feature *j* for sample *i*. Then the vector $y=(y_{1},\ldots ,y_{n}{)}^{⊺}$ represents the outcome, and the n×p matrix X represents the features. We focus on high-dimensional settings, where p≫n. To prepare the data for penalised regression, we standardise all features (zero mean, unit variance).

Given a predefined threshold for dichotomising the numerical outcome, samples with an outcome above this threshold are in class 1, and all other samples are in class 0. For each sample *i*, let $z_{i}$ indicate whether the numerical outcome $y_{i}$ is greater than the threshold *c*, or formally $z_{i}=I[y_{i}>c]$. Then the vector $z=(z_{1},\ldots ,z_{n}{)}^{⊺}$ represents the binary outcome. Since the transformation of y to z is non-invertible, y is at least as informative as z (but typically much more informative).

### 2.3. Logistic regression

We relate the binary outcome to the features through logistic regression:

$$\mathrm{logit}\left(P[z_{i}=1]\right)=\gamma_{0}+\sum_{j=1}^{p}\gamma_{j}x_{\mathrm{ij}},$$

where $\gamma_{0}$ is the unknown intercept, and $\left\{\gamma_{1},\ldots ,\gamma_{p}\right\}$ are the unknown slopes. The latter represent the effects of the features on the log-odds of the binary outcome. Given the estimated coefficients $\hat{\gamma }=({\hat{\gamma }}_{0},\ldots ,{\hat{\gamma }}_{p}{)}^{⊺}$, the predicted probabilities are $\hat{z}=({\hat{z}}_{1},\ldots ,{\hat{z}}_{n}{)}^{⊺}$, where ${\hat{z}}_{i}={\mathrm{logit}}^{-1}({\hat{\gamma }}_{0}+\sum_{j=1}^{p}{\hat{\gamma }}_{j}x_{\mathrm{ij}})$. For logistic regression, we use the logistic deviance as loss function:

$$L_{\log}(\gamma )=-2\sum_{i=1}^{n}\left\{z_{i}\log({\hat{z}}_{i})+(1-z_{i})\log(1-{\hat{z}}_{i})\right\},$$

which tends to zero if the predicted probabilities $\hat{z}$ approach 1 for positives $\left(z_{i}=1\right)$ and 0 for negatives $\left(z_{i}=0\right)$.

### 2.4. Linear regression

We relate the numerical outcome to the features through linear regression:

$$E[y_{i}]={\text{β}}_{0}+\sum_{j=1}^{p}{\text{β}}_{j}x_{\mathrm{ij}}\;,$$

where ${\text{β}}_{0}$ is the unknown intercept, and $\left\{{\text{β}}_{1},\ldots ,{\text{β}}_{p}\right\}$ are the unknown slopes. The latter represent the effects of the features on the numerical outcome. Given the estimated coefficients $\hat{\text{β}}=({\hat{\text{β}}}_{0},\ldots ,{\hat{\text{β}}}_{p}{)}^{⊺}$, the predicted values are $\hat{y}=({\hat{y}}_{1},\ldots ,{\hat{y}}_{n}{)}^{⊺}$, where ${\hat{y}}_{i}={\hat{\text{β}}}_{0}+\sum_{j=1}^{p}{\hat{\text{β}}}_{j}x_{\mathrm{ij}}$. For linear regression, we use the mean squared error as loss function:

$$L_{\mathrm{lin}}(\text{β})=\frac{1}{n}\sum_{i=1}^{n}(y_{i}-{\hat{y}}_{i}{)}^{2},$$

which tends to zero if the predicted values $\hat{y}$ approach the numerical outcomes y.

### 2.5. Parameter regularisation

We estimate the logistic and linear regression models by penalised maximum likelihood using lasso $\left(L_{1}\right)$ or ridge $\left(L_{2}\right)$ regularisation, which are generalised by elastic net regularisation [33]. Following the notation from [10], the penalties for logistic and linear regression are equal to

$$\begin{array}{rl} P_{\log}(\gamma |\lambda_{0},\alpha ) & =\lambda_{0}\sum_{j=1}^{p}\left(1-\alpha \right)\frac{\gamma_{j}^{2}}{2}+\alpha \left|\gamma_{j}\right|,\\ P_{\mathrm{lin}}(\text{β}|\lambda_{1},\alpha ) & =\lambda_{1}\sum_{j=1}^{p}\left(1-\alpha \right)\frac{{\text{β}}_{j}^{2}}{2}+\alpha \left|{\text{β}}_{j}\right|, \end{array}$$

where $\lambda_{0}$ and $\lambda_{1}$ are the regularisation parameters ( $\lambda_{0}\geq 0$, $\lambda_{1}\geq 0$), and α is the elastic net mixing parameter (0≤α≤1). The elastic net penalty collapses to the lasso or ridge penalty if α equals 1 or 0, respectively. We use the lasso penalty to estimate sparse models and the ridge penalty to estimate dense models, but it would also be possible to select α by tuning or combine multiple α by stacking [24].

The penalised loss functions for logistic and linear regression are the sums of the respective loss and penalty functions:

$$\begin{array}{rl} M_{\log}(\gamma |\lambda_{0},\alpha ) & =L_{\log}(\gamma )+P_{\log}(\gamma |\lambda_{0},\alpha )\;,\\ M_{\mathrm{lin}}(\text{β}|\lambda_{1},\alpha ) & =L_{\mathrm{lin}}(\text{β})+P_{\mathrm{lin}}(\text{β}|\lambda_{1},\alpha )\;. \end{array}$$

Given an elastic net mixing parameter α and the regularisation parameters $\lambda_{0}$ and $\lambda_{1}$, we can estimate the coefficients γ and β.

### 2.6. Model combination

We aim to improve the predicted probabilities from penalised logistic regression by accounting for the predicted values from penalised linear regression. Since the predicted values from linear regression are unbounded real numbers, we transform them to the unit interval via the Gaussian cumulative distribution function:

$$\Phi ({\hat{y}}_{i}|\mu =c,\sigma^{2})=\frac{1}{\sqrt{2\pi \sigma^{2}}}\int_{t=-\infty }^{{\hat{y}}_{i}}\exp\left\{-\frac{(t-c{)}^{2}}{2\sigma^{2}}\right\}dt\;,$$

where *μ* is the mean ( μ=c) and $\sigma^{2}$ is an optimisable variance ( $\sigma^{2}\geq 0$). This corresponds to the probit link, one of the two most common link functions for binary regression, with a fixed mean parameter for the threshold and a free variance parameter for calibration. The crucial difference to probit regression is that we do not model the *binary* outcome and transform the linear predictor to predicted probabilities but that we model the *numerical* outcome and transform predicted values to predicted probabilities. If the predicted value ${\hat{y}}_{i}$ is greater than the threshold *c*, the probability $\Phi ({\hat{y}}_{i}|\mu =c,\sigma^{2})$ is greater than 0.5. The variance $\sigma^{2}$ calibrates the probabilities: these diverge to 0 and 1 as $\sigma^{2}$ decreases, and converge to 0.5 as $\sigma^{2}$ increases. Intuitively, this transformation ‘confidently’ assigns samples to classes if $\sigma^{2}$ is small and ‘hesitantly’ if $\sigma^{2}$ is large.

For each sample *i*, we combine the predicted probability ${\hat{z}}_{i}$ from logistic regression and the predicted value ${\hat{y}}_{i}$ from linear regression:

$${\hat{p}}_{i}=(1-\pi ){\hat{z}}_{i}+\pi \Phi \left({\hat{y}}_{i}|\mu =c,\sigma^{2}\right),$$

where π is an optimisable weight ( 0≤π≤1). The weighting provides a compromise between the probabilities from penalised logistic regression and the calibrated probabilities from penalised linear regression. By construction, the combined values $\hat{p}=({\hat{p}}_{1},\ldots ,{\hat{p}}_{n}{)}^{⊺}$ are interpretable as probabilities. As the weight π increases, the contribution of logistic regression decreases, and the contribution of linear regression increases. The combined predicted probability ${\hat{p}}_{i}$ is completely determined by logistic or linear regression if π equals 0 or 1, respectively.

Again, we use the logistic deviance as loss function:

$$L_{\mathrm{com}}(\pi ,\sigma^{2})=-2\sum_{i=1}^{n}\left\{z_{i}\log({\hat{p}}_{i})+(1-z_{i})\log(1-{\hat{p}}_{i})\right\},$$

which tends to zero if the predicted probabilities $\hat{p}$ approach 1 for positives $\left(z_{i}=1\right)$ and 0 for negatives $\left(z_{i}=0\right)$.

In short, we combine the predicted probabilities from logistic regression ( $\hat{z}$) and the predicted values from linear regression ( $\hat{y}$) to the predicted probabilities $\hat{p}$, and we propose to interpret these combined predicted probabilities.

### 2.7. Parameter optimisation

We fix the elastic net mixing parameter α, tune the regularisation parameters $\lambda_{0}$ and $\lambda_{1}$, estimate the coefficients γ and β, and then estimate the weight parameter π and the scale parameter $\sigma^{2}$:

- tuning $\lambda_{0}$ and $\lambda_{1}$: We generate two sequences of 100 decreasing values for $\lambda_{0}$ and $\lambda_{1}$, with the largest values ( →∞) yielding empty models, and the smallest values ( →0) yielding full models. In *k*-fold cross-validation, we split the samples into *k* folds, repeatedly estimate the coefficients with *k*−1 included folds, and predict the outcomes for the excluded fold. In each iteration, we estimate the coefficients by minimising the penalised loss functions $M_{\log}(\gamma |\lambda_{0},\alpha )$ and $M_{\mathrm{lin}}(\text{β}|\lambda_{1},\alpha )$ with respect to γ or β, respectively, via coordinate descent along the regularisation path [10]. After the last iteration, we tune the regularisation parameters $\lambda_{0}$ and $\lambda_{1}$ to minimise the loss functions $L_{\log}(\gamma )$ and $L_{\mathrm{lin}}(\text{β})$.
- estimating γ, β, π and $\sigma^{2}$: Given the tuned regularisation parameters ${\hat{\lambda }}_{0}$ and ${\hat{\lambda }}_{1}$, we re-estimate the coefficients by minimising $M_{\log}(\gamma |{\hat{\lambda }}_{0},\alpha )$ and $M_{\mathrm{lin}}(\text{β}|{\hat{\lambda }}_{1},\alpha )$ with respect to γ and β. With the estimated coefficients $\hat{\gamma }$ and $\hat{\text{β}}$, we calculate the fitted probabilities $\hat{z}$ from logistic regression and the fitted values $\hat{y}$ from linear regression. To combine them, we estimate the weight and scale parameters by numerically minimising the loss function $L_{\mathrm{com}}(\pi ,\sigma^{2})$ with respect to π and $\sigma^{2}$.

This optimisation procedure first addresses penalised logistic ( $\lambda_{0}$, γ) and linear ( $\lambda_{1}$, β) regression separately, and then addresses their combination ( π, $\sigma^{2}$). Alternatively, we might use the expectation-maximisation (em) algorithm to iteratively estimate {γ,β} and $\left\{\pi ,\sigma^{2}\right\}$. In contrast to the em approach, our two-stage approach has practical advantages: the processing time is only slightly longer than for logistic and linear regression together, $\hat{\gamma }$ and $\hat{\text{β}}$ are interpretable as estimated effects of the features on the log-odds of the binary outcome or on the identity of the numerical outcome, respectively, and the local minima problem does not affect the estimation of the coefficients.

## 3. Simulation

### 3.1. Motivation

In this simulation study, we empirically show that combined regression outperforms not only logistic regression but also ‘calibrated linear regression’ at predicting dichotomised outcomes.

Logistic regression and calibrated linear regression are special cases of the proposed combined regression (with π=0 or π=1, respectively). While logistic regression requires the binary outcome and returns predicted probabilties, calibrated linear regression requires the numerical outcome and returns predicted values transformed to predicted probabilities.

We illustrate in four examples why the proposed combined regression – combining predicted probabilities from logistic regression and predicted values from linear regression – is suitable for predicting dichotomised outcomes.

### 3.2. Data generating process

Let *n* denote the sample size and let *p* denote the number of features. This is our process for generating features, effects and outcomes:

- features: Let $x_{\mathrm{ij}}$ represent feature *j* for sample *i*, for any *j* in {1,…,p} and any *i* in {1,…,n}. Simulating all values from a standard Gaussian distribution ( $x_{\mathrm{ij}}∼n(\mu =0,\sigma^{2}=1)$), we obtain the n×p feature matrix X.
- effects: Let ${\text{β}}_{j}$ represent the effect of feature *j*, for any *j* in {1,…,p}. Simulating all effects from a mixture distribution of a Bernoulli trial with success probability 5% and a standard Gaussian distribution ( ${\text{β}}_{j}∼b(n=1,\pi =0.05)\times n(\mu =0,\sigma^{2}=1)$), we obtain the *p*-dimensional vector $\text{β}=({\text{β}}_{1},\ldots ,{\text{β}}_{p}{)}^{⊺}$. While around 95% of the features have no effects, around 5% of the features have negative or positive effects of different sizes ( $\sum_{j=1}^{p}I[{\text{β}}_{j}\neq 0]/p\approx 0.05$).
- linear predictors: Let $\eta_{i}$ represent the linear predictor for sample *i*, for any *i* in {1,…,n}. Calculating all linear predictors from the effects and the features ( $\eta_{i}=\sum_{j=1}^{p}{\text{β}}_{j}x_{\mathrm{ij}}$), we obtain the *n*-dimensional vector $\eta =(\eta_{1},\ldots ,\eta_{n}{)}^{⊺}$.
- error terms: Let $\epsilon_{i}$ represent the error term for sample *i*, for any *i* in {1,…,n}. Simulating all error terms from a standard Gaussian distribution ( $\epsilon_{i}∼n(\mu =0,\sigma^{2}=1)$), we obtain the *n*-dimensional vector $\epsilon =(\epsilon_{1},\ldots ,\epsilon_{n}{)}^{⊺}$.
- outcomes: Let $y_{i}$ and $z_{i}$ represent the numerical or binary outcome of sample *i*, respectively, for any *i* in {1,…,n}. In each example, the numerical outcome depends on the linear predictor and the error term in a different way (see below). In all examples, the binary outcome $z_{i}$ indicates whether the numerical outcome $y_{i}$ is greater than the threshold zero $\left(z_{i}=I[y_{i}>0\right]$). The corresponding *n*-dimensional vectors are $y=(y_{1},\ldots ,y_{n}{)}^{⊺}$ and $z=(z_{1},\ldots ,z_{n}{)}^{⊺}$.

### 3.3. Examples

We provide one representative example where calibrated linear regression should outperform logistic regression, and three illustrative examples where logistic regression should outperform calibrated linear regression. In each example, the equation holds for any *i* in {1,…,n}.

1. standard setting: The numerical outcome equals the sum of the linear predictor and the error term.
$$y_{i}=\eta_{i}+\epsilon_{i}\;.$$
2. latent binary variable: The numerical outcome is clustered around a negative or positive value, depending on whether the linear predictor is below or above the threshold, respectively.
$$y_{i}=\left\{ \begin{array}{ll} -2+\epsilon_{i} & \;\mathrm{if}\;\eta_{i}<0\\ +2+\epsilon_{i} & \;\mathrm{if}\;\eta_{i}>0\;. \end{array}\right.$$
3. asymmetric relationship: The numerical outcome is not linearly related to the linear predictor but with a square-root below the threshold and a square above the threshold.
$$y_{i}=\left\{ \begin{array}{ll} -\sqrt{|\eta_{i}+\epsilon_{i}|} & \;\mathrm{if}\;\eta_{i}<0\\ +(\eta_{i}+\epsilon_{i}{)}^{2} & \;\mathrm{if}\;\eta_{i}>0\;. \end{array}\right.$$
4. presence of outliers: The numerical outcome usually equals the sum of the linear predictor and the error term, but rarely there is contamination by a large negative or a large positive number.
$$y_{i}=\left\{ \begin{array}{ll} \eta_{i}+\epsilon_{i} & \;\mathrm{with}\;P=95\%\\ \eta_{i}+\epsilon_{i}-1.5||\eta |{|}_{\infty } & \;\mathrm{with}\;P=2.5\%\\ \eta_{i}+\epsilon_{i}+1.5||\eta |{|}_{\infty } & \;\mathrm{with}\;P=2.5\%\;, \end{array}\right.$$
  where the infinity norm $||\eta |{|}_{\infty }$ returns the largest absolute value of $\eta =(\eta_{1},\ldots ,\eta_{n}{)}^{⊺}$.

### 3.4. Hold-out method

As there is no restriction on the sample size for simulated data, we simulate data for $n_{0}=100$ training samples but $n_{1}$=10,000 testing samples ( $n=n_{0}+n_{1}=$ 10,100) in each repetition of the hold-out method. Using *p* = 500 features, we obtain a high-dimensional setting because the number of features is much larger than the number of training samples ( $p\gg n_{0}$). After estimating the parameters of the three regression models with the 100 training samples, we predict the binary outcome for the 10,000 testing samples and compare the predicted probabilities ( $0\leq {\hat{p}}_{i}\leq 1$) with the observed classes ( $z_{i}=0$ or $z_{i}=1$).

### 3.5. Predictive performance

**Figure 2.**
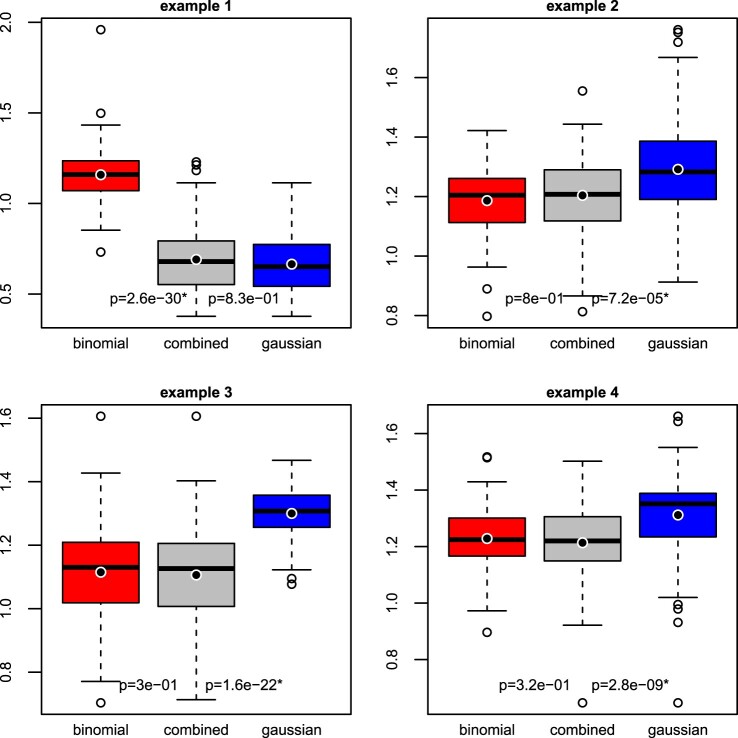
Out-of-sample logistic deviance (lower = better) from logistic regression (‘binomial’), combined regression, and calibrated linear regression (‘gaussian’), in four simulation settings. The black point added to the box plot represents the mean. A *p*-value with an asterisk indicates that the decrease in logistic deviance from logistic (left) or calibrated linear (right) to combined regression is statistically significant (one-sided Wilcoxon signed-rank test, Bonferroni-adjusted 5% significance level).

For each example in Section 3.3, we performed 100 repetitions of the hold-out method (i.e. simulating 100 sets of training and testing data). Figure 2 summarises the distributions of out-of-sample logistic deviances from logistic regression, calibrated linear regression, and combined regression, each under lasso regularisation. We tested whether combined regression leads to a significantly lower logistic deviance than logistic regression and calibrated linear regression, using the one-sided Wilcoxon signed-rank test.

We find that combined regression is significantly more predictive than logistic regression in the first example and significantly more predictive than calibrated linear regression in the other examples, at the Bonferroni-adjusted 5% level (*p*-value ≤0.05/8). Thus, combined regression is highly predictive because it combines the advantages of linear regression (efficiency) and logistic regression (robustness).

## 4. Application

The Montreal Cognitive Assessment (moca) is a screening tool for mild cognitive impairment (mci) [21]. Although the total moca score is a discrete numerical variable ranging from 0 to 30, researchers often model a binary variable indicating the absence or presence of cognitive impairment. For example, Fullard *et al.* [11] use Cox proportional hazards regression to predict the conversion time to mci, and Caspell-Garcia *et al*. [1] use logistic regression to predict mci, given the commonly accepted definition of mci as moca ≤25. Identifying patients at risk of cognitive impairment is important to develop measures for early intervention and prevention, such as cognitive training and physical exercise programmes. Here, we predict cognitive impairment from clinical features, analysing data from a longitudinal cohort study, the Parkinson's Progression Markers Initiative (ppmi) [17].

- features: We extracted the features from the curated baseline data. While the raw data include several hundred unfiltered variables in the categories ‘subject characteristics’, ‘biospecimen’, ‘digital sensor’, ‘enrolment’, ‘imaging’, ‘medical history’, ‘motor assessment’, ‘non-motor assessment’, and ‘remote data collection’, the curated data include 130 relevant variables, either selected or derived from the raw data (Supplementary Table A1). The proportion of missing data is approximately 3%.
- outcomes: We extracted the outcomes from the curated follow-up data, which cover the clinical visits after approximately one, two and three years. The total moca score is available for 390, 373 and 363 patients, indicating cognitive impairment (moca ≤25) for 34.4%, 32.4% and 32.2% of the patients, respectively. The apparent improvement likely results from non-random missingness, measurement variation, and training effects after repeated participation in cognitive assessments.

Our objective is to predict from clinical features at baseline which patients will have cognitive impairment after one, two or three years. While logistic regression only exploits the binary outcome of interest ‘total moca score ≤25 versus ≥26’, combined regression also exploits the underlying numerical outcome ‘total moca score’ to predict this probability.

We first imputed missing values in the feature matrix by chained random forests with predictive mean matching (R package missRanger) and then replaced categorical variables by dummy variables. Instead of imputing the missing values once and analysing one imputed data set (‘single imputation’), we imputed the missing values ten times and analysed each imputed data set separately (‘multiple imputation’).

For each imputed data set, we estimated the predictive performance of logistic and combined regression by nested cross-validation, with an internal loop for training and validation and an external loop for testing. In this unbiased evaluation, we split the samples into five folds, repeatedly train and validate the models with four folds, and test the models with the other fold. To obtain comparable performance estimates, we used the same 5 external and the same 10 internal folds for logistic and combined regression.

Algorithm 1 includes the high-level pseudocode for multiple imputation and nested cross-validation. In all comparisons, we used either lasso or ridge regularisation for both logistic and combined regression. We then examined the percentage change in cross-validated logistic deviance from logistic to combined regression (Supplementary Table A2). For both penalties ( $L_{1},L_{2}$) and all years (1, 2, 3), we observe an improvement for most imputations (8/10 or 10/10). This improvement also holds for other evaluation metrics, including the misclassification rate and the areas under the receiver operating characteristic and precision-recall curves (Supplementary Table A3).


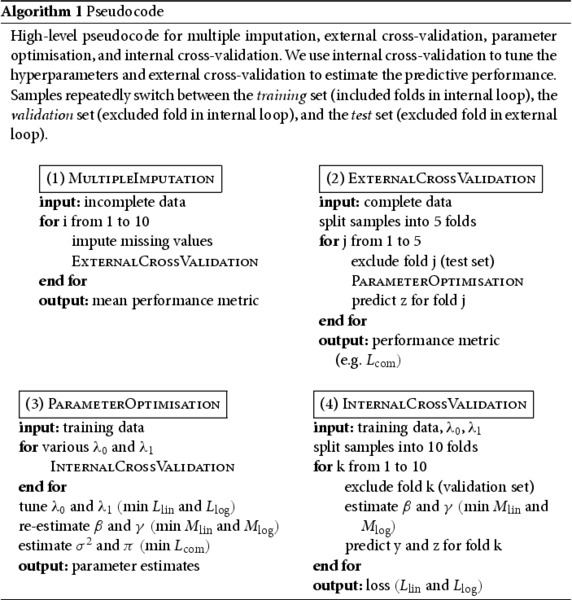


We used the multi-split approach from [31] to test the prediction error difference between logistic and combined regression. First, we randomly split the samples 50 times into 80% for training and validation, and 20% for testing. Then, for each split, we calculated the squared deviance residuals, whose mean equals the logistic deviance, and compared the paired residuals from logistic and combined regression with the one-sided Wilcoxon signed-rank test. Finally, we calculated the median *p*-value from the 50 splits, which maintains the type I error rate [31]. For each penalty ( $L_{1},L_{2}$), each year (1, 2, 3), and each imputation (1-10), the median *p*-value is significant at the 5% level (Supplementary Table A2). Therefore, combined regression leads to significantly better predictions than logistic regression. In this application, however, combined regression does not lead to significantly better prediction than calibrated linear regression (i.e. combined regression with zero weight for the logistic part). Here, two ensemble learning methods (random forest, gradient boosting) perform worse than ridge and lasso regression (Supplementary Table A4).

**Figure 3.**
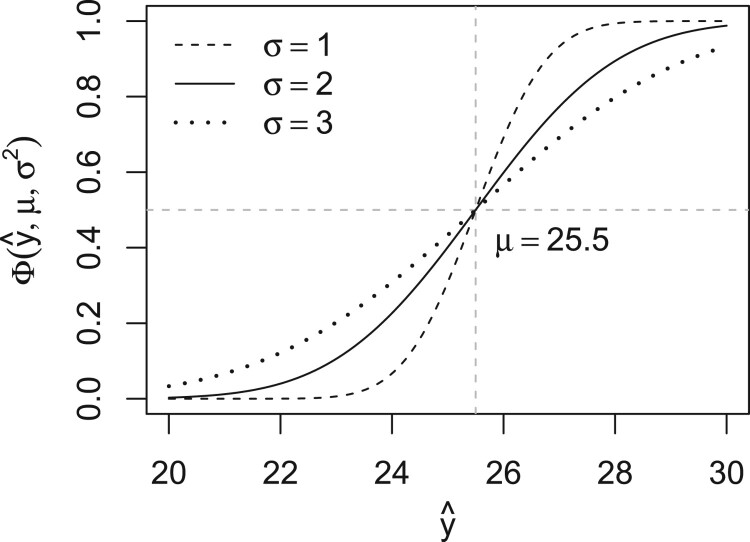
Transformation of predicted values (*x*-axis) to calibrated probabilities (*y*-axis) via the Gaussian cumulative distribution function with mean *μ* and variance $\sigma^{2}$. Predicted values above *μ* (vertical line) imply probabilities above 0.5 (horizontal line). While the mean *μ* equals the threshold *c*, we need to estimate the variance $\sigma^{2}$. The probabilities tend to 0 or 1 under small variances and to 0.5 under large variances.

**Figure 4.**
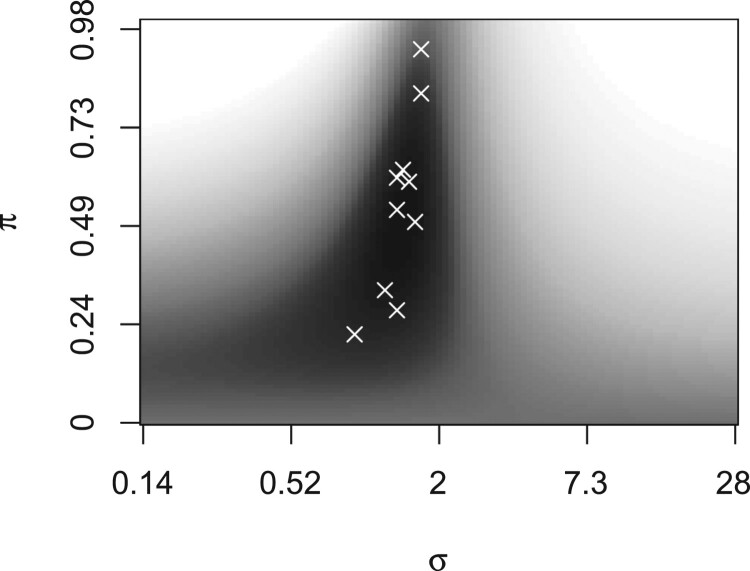
Logistic deviance given weight π (*y*-axis) and standard deviation σ (*x*-axis). The region with the lowest mean loss (dark) contains the selected tuning parameters (white crosses). Logistic regression obtains full weight if π equals 0 (bottom), and linear regression if π equals 1 (top). The latter renders predicted probabilities around 0 and 1 if σ is small (left) and around 0.5 if σ is large (right).

To examine weighting and scaling, we refitted combined regression to all folds. Depending on the penalty ( $L_{1},L_{2}$), the year (1, 2, 3), and the imputation (1-10), we estimated weights ( π) between 0.20 and 1.00 and variances ( $\sigma^{2}$) between $0.16^{2}$ and $1.70^{2}$ (Supplementary Table A2). Together, these estimates determine the combination of the predicted probabilities from logistic and linear regression. Figure 3 shows the transformation of predicted values from linear regression to calibrated probabilities, and Figure 4 shows the mean loss (for predicting the first-year outcome under lasso regularisation) at different combinations of weights and variances, where the mean is taken over the 10 imputations.

## 5. Discussion

We have proposed an approach for predicting dichotomised outcomes from high-dimensional data. Combining predicted probabilities from penalised logistic regression and predicted values from penalised linear regression, it achieves a high predictive performance, as shown by simulation and application. The general applicability includes biomedical prediction problems with clinically relevant thresholds.

Ideally, the threshold for dichotomisation is commonly established and splits the samples into two biologically relevant groups. If there is no practical or theoretical justification for setting the threshold equal to a specific value, the need for a probabilistic interpretation is questionable. Special care is required for data-dependent thresholds, because the same criterion typically leads to different thresholds in different data sets, and searching for the ‘optimal’ threshold typically leads to model overfitting.

Our approach integrates numerical information into binary classification, by first modelling binary and numerical outcomes separately and then combining the (calibrated) probabilities. This is related to transforming classifier scores to calibrated probabilities [19,25,32]. Given a threshold and predictions of the numerical outcome, we provide a probabilistic classification. Our aim is an interpretable combination of logistic and linear regression, but we recognise that non-parametric methods for mapping scores onto probabilities might improve the predictive performance.

Instead of applying linear regression on the numerical outcome and transforming the predicted values to probabilities, we could transform the numerical outcome to probabilities and apply logistic regression on the probabilities. Such an approach has previously been developed for low-dimensional settings [14,29]. However, due to the iteration between estimating the nuisance parameter and estimating the coefficients, an extension to high-dimensional settings would be computationally expensive. We estimate them separately but recognise that a simultaneous approach might provide superior performance.

Only the binomial distribution supports binary outcomes, but different distributions support quantitative outcomes. We chose the Gaussian distribution for modelling the quantitative outcome and for transforming predicted values to probabilities. This distribution is supported on the whole real line and has two parameters for thresholding and calibration. It is possible to use different distributions for modelling the observed outcomes or transforming the predicted outcomes. For the first, we could model counts with the Poisson or the negative binomial distribution. For the latter, we could increase flexibility with the three-parameter log-normal distribution [29] or the skew normal distribution.

Since the numerical outcome is normally more informative than the binary outcome, it is not surprising that modelling the numerical outcome next to the binary outcome improves the predictions of the binary outcome. A more important result is that modelling the numerical and the binary outcomes together can provide better predictions than modelling only the numerical outcome. Similarly, numerical features and binary transformations of the same features can be more predictive together than alone [22].

The proposed approach combines the predicted probabilities from logistic regression and the predicted values from linear regression, leaving their estimated coefficients untouched. If the aim was to merge the estimated coefficients from logistic and linear regression into a single set of estimated coefficients, one could use bivariate regression by stacked generalisation for the binary and the numerical outcome [23]. However, this would make the combination of predicted probabilities and predicted values less interpretable.

Although this study focuses on dichotomised outcomes, it is not our intention to advocate dichotomisation. Numerical outcomes should not be binarised, unless there is a strong reason to the contrary. On this condition, we recommend to exploit the binary *and* the numerical outcome. For binary classification in high-dimensional settings, the proposed approach combines both sources of information.

## 6. Conclusion

For predicting *numerical* outcomes, we suggest to use penalised linear regression to obtain predicted values. For *natural binary* outcomes, we suggest to use penalised logistic regression to obtain predicted probabilities. And for *artificial binary* outcomes (also known as dichotomised outcomes), we propose to combine penalised linear and logistic regression to obtain predicted probabilities.

## Reproducibility

The R package cornet includes the code for the simulation and the application (https://cran.r-project.org/package=cornet). We obtained our results using R 4.3.0 with cornet 0.0.8 on a physical machine (aarch64-apple-darwin20, macOS Ventura 13.4). Data used in the preparation of this article were obtained from the Parkinson's Progression Markers Initiative (ppmi) database (https://www.ppmi-info.org/data).

## Supplementary Material

Supplemental Material

## References

[1] C. Caspell-Garcia, T. Simuni, D. Tosun-Turgut, I. Wu, Y. Zhang, M. Nalls, A. Singleton, L.A. Shaw, J.-H. Kang, J.Q. Trojanowski, A. Siderowf, C. Coffey, S. Lasch, D. Aarsland, D. Burn, L.M. Chahine, A.J. Espay, E.D. Foster, K.A. Hawkins, I. Litvan, I. Richard, and D. Weintraub, Multiple modality biomarker prediction of cognitive impairment in prospectively followed de novo Parkinson disease, PLoS One 12 (2017), Article ID e0175674. doi: 10.1371/journal.pone.0175674 PMC5435130 28520803

[2] J. Cohen, The cost of dichotomization, Appl. Psychol. Meas. 7 (1983), pp. 249–253. doi: 10.1177/014662168300700301

[3] N.V. Dawson and R. Weiss, Dichotomizing continuous variables in statistical analysis: A practice to avoid, Med. Decis. Making 32 (2012), pp. 225–226. doi: 10.1177/0272989X12437605 22457338

[4] A.R. de Leon and B. Wu, Copula-based regression models for a bivariate mixed discrete and continuous outcome, Stat. Med. 30 (2011), pp. 175–185. doi: 10.1002/sim.4087 20963753

[5] M. de Paula and C.A.R. Diniz, Generalized linear regression models incorporating original outcome distributions, Commun. Stat. – Theory Methods 45 (2016), pp. 5762–5786. doi: 10.1080/03610926.2014.948726

[6] A. Dupuy and D. Nassar, Dichotomization of primary outcomes serves external validity, J. Invest. Dermatol. 134 (2014), pp. 266–267. doi: 10.1038/jid.2013.258 23756709

[7] D.P. Farrington and R. Loeber, Some benefits of dichotomization in psychiatric and criminological research, Crim. Behav. Ment. Health 10 (2000), pp. 100–122. doi: 10.1002/cbm.349

[8] V. Fedorov, F. Mannino, and R. Zhang, Consequences of dichotomization, Pharm. Stat. 8 (2009), pp. 50–61. doi: 10.1002/pst.331 18389492

[9] G.M. Fitzmaurice and N.M. Laird, Regression models for a bivariate discrete and continuous outcome with clustering, J. Am. Stat. Assoc. 90 (1995), pp. 845–852. doi: 10.2307/2291318

[10] J. Friedman, T. Hastie, and R. Tibshirani, Regularization paths for generalized linear models via coordinate descent, J. Stat. Softw. 33 (2010), pp. 1–22. doi: 10.18637/jss.v033.i01 (glmnet). 20808728 PMC2929880

[11] M.E. Fullard, B. Tran, S.X. Xie, J.B. Toledo, C. Scordia, C. Linder, R. Purri, D. Weintraub, J.E. Duda, L.M. Chahine, and J.F. Morley, Olfactory impairment predicts cognitive decline in early Parkinson's disease, Parkinsonism Relat. Disord. 25 (2016), pp. 45–51. doi: 10.1016/j.parkreldis.2016.02.013 26923521 PMC4825674

[13] F.E. Harrell, *General aspects of fitting regression models – avoiding categorization; ordinal logistic regression*, in *Regression Modeling Strategies*, Springer, Cham, 2015, pp. 311–325. doi: 10.1007/978-3-319-19425-7

[14] S. Heritier and E. Ronchetti, Robust binary regression with continuous outcomes, Can. J. Stat. 32 (2004), pp. 239–249. doi: 10.2307/3315927

[15] O. Kuss, The danger of dichotomizing continuous variables: A visualization, Teach. Stat. 35 (2013), pp. 78–79. doi: 10.1111/test.12006

[16] R.C. MacCallum, S. Zhang, K.J. Preacher, and D.D. Rucker, On the practice of dichotomization of quantitative variables, Psychol. Methods 7 (2002), pp. 19–40. doi: 10.1037/1082-989X.7.1.19 11928888

[17] K. Marek, D. Jennings, S. Lasch, A. Siderowf, and C. Tanner, The Parkinson Progression Marker Initiative (PPMI), Prog. Neurobiol. 95 (2011), pp. 629–635. doi: 10.1016/j.pneurobio.2011.09.005 21930184 PMC9014725

[18] B.K. Moser and L.P. Coombs, Odds ratios for a continuous outcome variable without dichotomizing, Stat. Med. 23 (2004), pp. 1843–1860. doi: 10.1002/sim.1776 15195319

[19] M.P. Naeini and G.F. Cooper, Binary classifier calibration using an ensemble of piecewise linear regression models, Knowl. Inf. Syst. 54 (2018), pp. 151–170. doi: 10.1007/s10115-017-1133-2 29606784 PMC5875942

[20] O. Naggara, J. Raymond, F. Guilbert, D. Roy, A. Weill, and D.G. Altman, Analysis by categorizing or dichotomizing continuous variables is inadvisable: An example from the natural history of unruptured aneurysms, AJNR Am. J. Neuroradiol. 32 (2011), pp. 437–440. doi: 10.3174/ajnr.A2425 21330400 PMC8013096

[21] Z.S. Nasreddine, N.A. Phillips, V. Bédirian, S. Charbonneau, V. Whitehead, I. Collin, J.L. Cummings, and H. Chertkow, The Montreal Cognitive Assessment, MoCA: A brief screening tool for mild cognitive impairment, J. Am. Geriatr. Soc. 53 (2005), pp. 695–699. doi: 10.1111/j.1532-5415.2005.53221.x 15817019

[22] A. Rauschenberger, I. Ciocănea-Teodorescu, M.A. Jonker, R.X. Menezes, and M.A. van de Wiel, Sparse classification with paired covariates, Adv. Data Anal. Classif. 14 (2020), pp. 571–588. doi: 10.1007/s11634-019-00375-6 (palasso).

[23] A. Rauschenberger and E. Glaab, Predicting correlated outcomes from molecular data, Bioinform. 37 (2021), pp. 3889–3895. doi: 10.1093/bioinformatics/btab576 (joinet). PMC10186156 34358294

[24] A. Rauschenberger, E. Glaab, and M.A. van de Wiel, Predictive and interpretable models via the stacked elastic net, Bioinform. 37 (2021), pp. 2012–2016. doi: 10.1093/bioinformatics/btaa535 (starnet). PMC8336997 32437519

[25] J. Schwarz and D. Heider, GUESS: Projecting machine learning scores to well-calibrated probability estimates for clinical decision-making, Bioinform. 35 (2019), pp. 2458–2465. doi: 10.1093/bioinformatics/bty984 (CalibratR). 30496351

[26] Y. Shentu and M. Xie, A note on dichotomization of continuous response variable in the presence of contamination and model misspecification, Stat. Med. 29 (2010), pp. 2200–2214. doi: 10.1002/sim.3966 20812301

[27] D.L. Streiner, Breaking up is hard to do: The heartbreak of dichotomizing continuous data, Can. J. Psychiatry 47 (2002), pp. 262–266. doi: 10.1177/070674370204700307 11987478

[28] S. Suissa, Binary methods for continuous outcomes: A parametric alternative, J. Clin. Epidemiol. 44 (1991), pp. 241–248. doi: 10.1016/0895-4356(91)90035-8 1999683

[29] S. Suissa and L. Blais, Binary regression with continuous outcomes, Stat. Med. 14 (1995), pp. 247–255. doi: 10.1002/sim.4780140303 7724910

[30] R. Ulrich and M. Wirtz, On the correlation of a naturally and an artificially dichotomized variable, Br. J. Stat. Psychol. 57 (2004), pp. 235–251. doi: 10.1348/0007110042307203 15511306

[31] M.A. van de Wiel, J. Berkhof, and W.N. van Wieringen, Testing the prediction error difference between 2 predictors, Biostat. 10 (2009), pp. 550–560. doi: 10.1093/biostatistics/kxp011 19380517

[32] B. Zadrozny and C. Elkan, Transforming classifier scores into accurate multiclass probability estimates, in *Proceedings of the Eighth ACM SIGKDD International Conference on Knowledge Discovery and Data Mining*, 2002, pp. 694–699. doi: 10.1145/775047.775151

[33] H. Zou and T. Hastie, Regularization and variable selection via the elastic net, J. R. Stat. Soc., B: Stat. Methodol. 67 (2005), pp. 301–320. doi: 10.1111/j.1467-9868.2005.00503.x

