# Supplementary material for: Predicting dichotomised outcomes from high-dimensional data in biomedicine
Source: J Appl Stat. 2023 Jul 26;51(9):1756–71. doi: 10.1080/02664763.2023.2233057 (PMC11198132; doi:10.1080/02664763.2023.2233057)
Supplement: Supplemental Material [file CJAS_A_2233057_SM6864.pdf]

Appendix A. Supplementary Tables

The appendix includes additional information on the application (Tables A1, A2, A3 and A4). For reproducibility of the simulation and the application, see the vignettes of the R package cornet (https://github.com/rauschenberger/cornet, https://CRAN.R-project.org/package=cornet).

Table A1. Included variables from the PPMI curated baseline data (all variables except *apprdx*, *event_id*, *patno*, *site* and *symptom5_comment*). The variable *apprdx* indicates subjects with Parkinson's disease (code 1), and the variable *patno* identifies the subjects. For further information, see *Curated data cut - Baseline data (dictionary)*, *Curated data cut - Year 1-3 data (dictionary)*, and *Variable Definitions and Score Calculations* in the PPMI database.

ab_asyn: Ratio of CSF A-beta 1-42 to CSF Alpha-synuclein
abeta: CSF A-beta 1-42 (2016 assay)
age: Age at Baseline
age_cat: Categorical Age at Baseline
agediag: Age at PD Diagnosis
ageonset: Age at Symptom Onset
ai_caudate: Asymmetry Index (Caudate)
ai_putamen: Asymmetry Index (Putamen)
ai_striatum: Asymmetry Index (Striatum)
APOE: APOE Genotype
APOE e4: APOE Genotype - number of e4 alleles
asyn: CSF Alpha-synuclein (2016 assay)
bjlot: Benton Judgement of Line Orientation Score
caudate_l: Left caudate
caudate_r: Right caudate
con_caudate: Contralateral caudate
con_cdr: Contralateral count density ratio
con_putamen: Contralateral putamen
con_striatum: Contralateral striatum
domside: Side most affected at PD onset
duration: Duration of Disease since Diagnosis (Months)
educ: Categorical Education
educyrs: Years of Education
ess: Epworth Sleepiness Scale Score
ess_cat : Categorical Epworth Sleepiness Scale Score
fampd_new : Family History of PD - new categories
fampd_old: Family History of PD - original categories
gds: Geriatric Depression Scale Score
gds_cat: Categorical Geriatric Depression Scale
gen: Gender
hemo: CSF Hemoglobin (2016 assay)
hemo_above: Hemoglobin value above limit of detection
hemo_below : Hemoglobin value below limit of detection
hemohi: Categorical CSF hemoglobin
hi_caudate: Highest caudate measure
hi_putamen: Highest putamen measure
hi_striatum: Highest striatum measure
hisplat: Ethnicity
hvlt_discrimination: HVLT Discrimination Recognition
hvlt_immediaterecall: HVLT Immediate/Total Recall
hvlt_retention: HVLT Retention
hvltfprl : HVLT False Alarms
hvltrdly: HVLT Delayed Recall
hvltrec: HVLT Delayed Recognition
hy: Categorical Hoehn & Yahr
ips_caudate: Ipsliateral caudate
ips_cdr: Ipsliateral count density ratio
ips_putamen: Ipsilateral putamen
ips_striatum: Ipsilateral striatum
l_cdr: Left count density ratio
l_striatum: Left striatum
lns: Letter Number Sequencing Score
low_caudate: Lowest caudate measure
low_putamen: Lowest putamen measure
low_striatum: Lowest striatum measure
MAPT: MAPT Genotype
MAPT cat: Categorical MAPT Genotype
mean_caudate: Mean caudate measure
mean_putamen: Mean putamen measure
mean_striatum: Mean striatum measure
moca: MOCA Score (adjusted for education)
mseadlg: Modified Schwab & England ADL Score
nhy: Hoehn & Yahr Stage
np1anxs: MDS-UPDRS Part I Anxious Mood
np1apat: MDS-UPDRS Part I Apathy
np1cog: MDS-UPDRS Part I Cognitive Impairment
np1dds: MDS-UPDRS Part I Features of Dopamine Dysregulation Syndrome
np1dprs: MDS-UPDRS Part I Depressed Mood
np1fatg: MDS-UPDRS Part I Fatigue
np1hall: MDS-UPDRS Part I Hallucinations and Psychosis
pd_med_use: Use of PD Medications at the time of the Study Visit
ptau: CSF p-tau (2016 assay)
ptau_ab: Ratio of CSF p-tau to CSF A-beta 1-42
ptau_asyn: Ratio of CSF p-tau to CSF Alpha-synuclein
ptau_tau: Ratio of CSF p-tau to CSF t-tau
putamen_l: Left putamen
putamen_r: Right putamen
quip: QUIP Score
quip_any: Any QUIP disorder
quip_buy: QUIP disorder - Buying
quip_eat: QUIP disorder - Eating
quip_gamble: QUIP disorder - Gambling
quip_hobby: QUIP disorder - Hobbies
quip_pund : QUIP disorder - Punding
quip_sex: QUIP disorder - Sex
quip_walk: QUIP disorder - Walking or Driving
r_cdr: Right count density ratio
r_striatum: Right striatum
race: Race
rem: REM Sleep Behavior Disorder Questionnaire Score
rem_cat: Categorical REM Sleep Behavior Disorder
rem_q6: Categorical REM Sleep Behavior Disorder based on Question 6 only
rigidity: Total Rigidity Score
scopa: SCOPA-AUT Total Score
scopa_cv: SCOPA-AUT Cardiovascular Score
scopa_gi: SCOPA-AUT Gastrointestinal (GI) Score
scopa_pm: SCOPA-AUT Pupillomotor Score
scopa_sex : SCOPA-AUT Sexual Dysfunction Score
scopa_therm: SCOPA-AUT Thermoregulatory Score
scopa_ur: SCOPA-AUT Urinary Score
sdmtotal: Symbol Digit Modalities Score
sft: Semantic Fluency Total Score
SNCA_rs356181: SNCA rs356181 Genotype
SNCA_rs356181_cat: Categorical SNCA rs356181 Genotype
SNCA_rs3910105: SNCA rs3910105 Genotype
SNCA_rs3910105_cat: Categorical SNCA rs3910105 Genotype
stai: STAI Total Score
stai_state: STAI State Sub-score
stai_trait: STAI Trait Sub-score
symptom1 : Initial symptom (at diagnosis) - Resting Tremor
symptom2 : Initial symptom (at diagnosis) - Rigidity
symptom3 : Initial symptom (at diagnosis) - Bradykinesia
symptom4: Initial symptom (at diagnosis) - Postural Instability
symptom5: Initial symptom (at diagnosis) - Other
symptom6: Missing initial symptoms
tau: CSF t-tau (2016 assay)
tau_ab: Ratio of CSF t-tau to CSF A-beta 1-42
tau_asyn: Ratio of CSF t-tau to CSF Alpha-synuclein
td_pigd: TD/PIGD classification - new categories
td_pigd_old: TD/PIGD classification - original categories
tremor: Tremor Score
updrs1_score: MDS-UPDRS Part I Score
updrs2_score: MDS-UPDRS Part II Score
updrs3_score: MDS-UPDRS Part III Score
updrs_totscore: MDS-UPDRS Total Score
upsit: UPSIT Score
urate: Serum Uric Acid
vltanim: Semantic Fluency Score - Animal subscore
vltfruit: Semantic Fluency Score - Fruit subscore
vltveg: Semantic Fluency Score - Vegetable subscore

Table A2. Pooled results from 10 imputations. The row indicates the setting (eiher lasso or ridge penalty for both methods, 1st/2nd/3rd year). Combined regression outperforms logistic regression: best (δ_{min}) and worst (δ_{max}) percentage change in cross-validated deviance from logistic to combined regression, number of improvements (δ_{num}), minimum (p_{min}) and maximum (p_{max}) median p-value from multi-split approach. Combined regression exploits both binary and numerical information: minimum, median and maximum estimates for the weight (π) and scale (σ) parameters.

	δ_{min}	δ_{max}	δ_{num}	p_{min}	p_{max}	π_{min}	π_{med}	π_{max}	σ_{min}	σ_{med}	σ_{max}
lasso 1	-2.3	-0.7	10	0.006	0.021	0.22	0.56	0.93	0.94	1.40	1.70
lasso 2	-5.0	0.0	10	0.004	0.014	0.27	0.41	0.61	0.16	1.06	1.45
lasso 3	-4.6	-1.0	10	0.001	0.006	0.40	0.52	0.63	1.00	1.24	1.38
ridge 1	-4.4	-1.3	10	0.004	0.011	0.39	0.66	1.00	1.16	1.48	1.61
ridge 2	-2.5	0.7	8	0.009	0.031	0.20	0.26	0.49	0.16	0.28	1.53
ridge 3	-1.4	0.6	8	0.002	0.007	0.21	0.32	0.52	0.19	1.14	1.38

Table A3. Median percentage change in cross-validated performance metrics from logistic to combined regression (across 10 imputations). The row indicates the setting (either lasso or ridge penalty for both methods, 1st/2nd/3rd year), and the column indicates the metric (logistic deviance, MCR = misclassification rate, MSE = mean squared error, MAE = mean absolute error, ROC-AUC = area under the receiver operating characteristic curve, PR-AUC = area under the precision recall curve).

	Δdeviance	ΔMCR	ΔMSE	ΔMAE	ΔROC-AUC	ΔPR-AUC
lasso 1	-1.4	-2.7	-1.9	-5.7	+0.6	+0.2
lasso 2	-2.2	-5.2	-3.3	-8.3	+1.0	+0.6
lasso 3	-2.7	-6.9	-4.0	-9.0	+1.0	+0.1
ridge 1	-2.3	-2.9	-2.8	-6.7	+1.4	+0.8
ridge 2	-0.9	-5.7	-1.9	-6.6	+0.2	+0.1
ridge 3	-0.4	-1.5	-1.2	-6.8	-0.2	-0.2

Table A4. Median cross-validated logistic deviance (across 10 imputations) for the comparison with random forest (randomForest, default parameters) and gradient boosting (xgboost, default parameters, 500 boosting iterations). A decrease from logistic lasso regression to combined lasso regression or from logistic ridge regression to combined ridge regression means that the proposed approach improves the predictive performance. Similarly, it would be possible to improve random forest or gradient boosting by modelling not only the binary outcome but also the numerical outcome (and possibly also by hyperparameter tuning or probability calibration).

	year 1	year 2	year 3
logistic lasso regression	0.98	0.96	0.89
combined lasso regression	0.96	0.93	0.87
logistic ridge regression	0.97	0.97	0.88
combined ridge regression	0.95	0.96	0.88
randomForest	1.04	0.99	0.94
xgboost	1.58	1.51	1.27
